# Polyethylene Glycol 35 as a Perfusate Additive for Mitochondrial and Glycocalyx Protection in HOPE Liver Preservation

**DOI:** 10.3390/ijms21165703

**Published:** 2020-08-09

**Authors:** Arnau Panisello Rosello, Rui Teixeira da Silva, Carlos Castro, Raquel G. Bardallo, Maria Calvo, Emma Folch-Puy, Teresa Carbonell, Carlos Palmeira, Joan Roselló Catafau, René Adam

**Affiliations:** 1 Experimental Hepatic Ischemia-Reperfusion Unit, Institut d’Investigacions Biomèdiques de Barcelona (IIBB), Spanish National Research Council (CSIC)-IDIBAPS, CIBEREHD, 08036 Barcelona, Catalonia, Spain; arnau.panisello@iibb.csic.es (A.P.R.); rui.teixeira@iibb.csic.es (R.T.d.S.); emma.folch@iibb.csic.es (E.F.-P.); 2 Centre Hépato-Biliaire, AP-PH, Hôpital Paul Brousse, 94800 Villejuif, France; castrob@gmail.com (C.C.); rene.adam@aphp.fr (R.A.); 3 Center for Neuroscience and Cell Biology, Universidade Coimbra, 3000-370 Coimbra, Portugal; palmeira@ci.uc.pt; 4 Department of Cell Biology, Physiology and Immunology, Universitat de Barcelona, 08028 Barcelona, Catalonia, Spain; rgomezbardallo@ub.edu (R.G.B.); tcarbonell@ub.edu (T.C.); 5 Serveis Cientifico Tècnics, 08036-Campus Hospital Clínic, Universitat de Barcelona, 08919 Barcelona, Catalonia, Spain; mariacalvo@ub.edu

**Keywords:** polyethylene glycol 35 (PEG35), hydroxyethyl starch (HES), UW solution, IGL-1 solution, Belzer-MPS, HOPE, liver graft preservation

## Abstract

Organ transplantation is a multifactorial process in which proper graft preservation is a mandatory step for the success of the transplantation. Hypothermic preservation of abdominal organs is mostly based on the use of several commercial solutions, including UW, Celsior, HTK and IGL-1. The presence of the oncotic agents HES (in UW) and PEG35 (in IGL-1) characterize both solution compositions, while HTK and Celsior do not contain any type of oncotic agent. Polyethylene glycols (PEGs) are non-immunogenic, non-toxic and water-soluble polymers, which present a combination of properties of particular interest in the clinical context of ischemia-reperfusion injury (IRI): they limit edema and nitric oxide induction and modulate immunogenicity. Besides static cold storage (SCS), there are other strategies to preserve the organ, such as the use of machine perfusion (MP) in dynamic preservation strategies, which increase graft function and survival as compared to the conventional static hypothermic preservation. Here we report some considerations about using PEG35 as a component of perfusates for MP strategies (such as hypothermic oxygenated perfusion, HOPE) and its benefits for liver graft preservation. Improved liver preservation is closely related to mitochondria integrity, making this organelle a good target to increase graft viability, especially in marginal organs (e.g., steatotic livers). The final goal is to increase the pool of suitable organs, and thereby shorten patient waiting lists, a crucial problem in liver transplantation.

## 1. Introduction

For more than 100 years, a fascinating dream has been to keep organs “alive” outside the human body [1]. In the process of transplantation, the storage of organs in preservation solutions is a mandatory step for maintaining their quality and for the success of the transplantation [2,3]. For many years, the University of Wisconsin (UW) solution was considered as the gold standard [4,5]. However, other solutions containing (or not) oncotic agent in their compositions, such as Institut Georges Lopez 1 (IGL-1) [6], Celsior [7] and histidine-tryptophan-ketoglutarate (HTK) [8], are also commonly used for static cold storage (SCS) in clinical transplantation. UW preservation solution contains the oncotic agent hydroxyethyl starch (HES) [5], in contrast to IGL-1, which contains polyethylene glycol 35 (PEG35) [6] as a main component (along with other components). Other commercially available solutions used, such as Celsior and HTK [7,8], have no oncotic agent in their formulations. All of them are considered a good alternative to UW in liver transplantation according to data from the European Liver Transplant Registry (ELTR), albeit with some limitations for HTK [9].

PEGs are polymers of ethylene oxide with hydroxyl termini. They are neutral, water-soluble, non-toxic and non-immunogenic polymers, characterized by their three-dimensional conformation and their high flexibility [10,11]. The molecular weight (MW) of the molecule depends on the length of the HO-(CH2CH2O) n-CH2-CH2-OH chain, which confers them characteristics of density and high hydrophilicity. PEGs are negligibly synthetized in vivo, and their non-toxicity makes them FDA approved, being used for many purposes (industrial, alimentary, pharmacologic, etc.) [11].

Robinson firstly reported the relevance of oedema prevention by PEG [12,13,14]. Further studies demonstrated that PEG cannot cross membranes, thereby exerting an oncotic pressure that limits tissue edema without breaking the transmembrane ionic balance [14]. In 1976, Daniel and Wakerley [15] used 20 kDa PEG to demonstrate increased cell viability during the preservation at 4 °C of renal pig cells. In 1977, Ganote et al. [16] demonstrated that low molecular (6 kDa) PEG avoids cellular edema in heart rat tissue during the preservation, limiting the regions damaged by hypoxia. Based on this line of studies, the use of 8 kDa PEG permitted a 24 h preservation to be obtained in isolated hepatocytes, reducing tissue edema and cell mortality by preserving actin microfilaments and the microtubules integrity [17].

Investigations by Wicomb et al. [18] resulted in the modification of the cardiac preservation solution St. Thomas by adding PEG 20 (20 kDa PEG), demonstrating an improved preservation and an interest in the use of PEGs to a clinical setting. Further studies proved that higher molecular PEGs, such as the PEG 35 used in the IGL-1 solution, is protective for liver graft preservation [19], making it an effective alternative to UW (the gold standard) used in clinical liver transplantation [9].

IGL-1 is the unique PEG based solution routinely used in clinical liver transplantation, and it contains PEG35 at 1 g/L. The use of other solutions, such as SCOT containing PEG 20 (at 15 g/L), has been limited, with non-conclusive results. In fact, in 2019, Karam et al. [20] and previously in 2017, Adam et al. [21] described a large series of transplants performed at the Paul Brousse Hospital (France) that shows a higher incidence of early allograft dysfunction in HTK- and SCOT-preserved ECD livers as compared to other solutions (e.g., UW and IGL-1) and that this negatively affects graft survival [20,21]. This could be due to the lower molecular weight of PEG 20 used in SCOT solution, which requires its concentration to be increased to 15 g/L (as compared to 1 g/L for PEG 35 in IGL-1). This in turn increases viscosity which could not only hinder an efficient graft washout after organ recovery but also promote deleterious effects on endothelial shear stress in graft preservation processes. Moreover, the presence of a glucose SCOT solution could promote deleterious effects in preserved liver graft during SCS due to the generation of acidosis in oxygen deprivation conditions [22]. Other PEG-containing solutions are IGL-1, Polysol and IGL2 [6,23,24], as shown in Table 1.

With this in mind, a PEG35 rinse solution contributes to a more efficient washout of the graft. As this avoids the hyper-aggregation action of HES against red blood cells in recovered liver graft when UW solution is used, it also confers additional protection against reperfusion [11]. Moreover, the presence of PEG35 in a rinse solution provides significantly more mitochondrial protection as compared to a rinse solution without PEG35 [19].

More recently, it has been demonstrated that intravenous (i.v.) PEG35 administration is also protective against liver warm ischemia reperfusion injury [25]. This protection has been reviewed in other organs, such as pancreas in an inflammation model [26]. This protective effect was demonstrated in i.v. PEG35 pre-treatment in rats [26]. Several studies reported the efficiency of PEGs in upregulating cell-survival pathways in different organs [27,28], resulting in the protection of the mitochondria, prevention of radical oxygen species (ROS) formation, cell swelling and oedema, and preservation of the cellular membrane, among others. It is thought that, under conditions of low surface pressure, PEGs stabilize the cell membranes [29]. This effect is partly associated with the ability of PEGs to reduce the lipid molecular motion, which may cause denser packing of lipids and decrease the fluidity of the membrane [29].

PEGs have been shown to lower monolayer surface pressures and to increase the area per lipid at the “collapse point”, at which point glycerophospholipids are densely packaged. Conditions of hypothermia imposes lipid phase transitions in the membranes, which modify the conformation of the lipid packing. This pressure-lowering effect of PEG may be evident in membrane regions with saturated phospholipids and, to a lesser degree, on unsaturated acyl chains in membrane monolayers. Temperature (conditioning the ion strength) and pH of the environment are extremely important parameters, which have direct effects on the binding properties of PEG [29,30,31]. The precise mechanisms by which high molecular PEGs exert their liver cytoprotection still remain unclear, although Belzer and Southard suggested the relevance of underlying mechanisms 30 years ago [4,5]. They pointed out that “the mechanism by which PEG prevent cell swelling is not related to the osmotic or oncotic properties of the molecule but instead is apparently related to some “unknown interactions” between PEG and cells, an interaction that provides stability during hypothermic preservation of hepatocytes” [32].

It is important to note that the properties of PEGs contribute to the sustained maintenance of cold, which is a determinant parameter for graft integrity during conservation, as evidenced by several studies [33,34,35].

We have witnessed an increasing amount of evidence that points out to the crucial role of oxygen and mitochondria during ischemia. As a consequence, in the last decade, the hypothermic oxygenated perfusion (HOPE) has been implemented as a complementary tool to improve graft preservation [36,37,38,39]. This strategy combines the benefits of cold preservation conditions with the presence of a transient oxygen supply to the organ in a perfusion system, with the purpose to help the mitochondrial system to sustain a basic level of oxidative phosphorylation, which in turn will provide the organ with better conditions to face the unavoidable reperfusion [36,37,38,39]. In contrast to static preservation, the fluid dynamics that intrinsically occurs in any fluid-applying forces in a vascular section, as it happens in HOPE (or any kind of reperfusion), is responsible for generating the phenomenon known as shear stress [40]. Shear stress affects the surface of the section and hence the glycocalyx, the luminal thin monolayer of sugars that covers the blood vessel endothelia [41]. In this context, perfusates used in HOPE may play a determinant role in the associated mechano-transduction processes involved in shear stress [42].

The most commonly used perfusion solution for HOPE is a variation of the original SCS UW solution, the Belzer Machine Perfusion Solution (Belzer MPS) and its generics. As the same formulation is used throughout this article, we refer to them generically as Belzer MPS. This solution was designed for kidney MP and then extended to liver HOPE strategies [36,37,38,39]. However, the use of Belzer MPS–like solutions in liver MP has some limitations. First, it contains HES that contributes to increasing the viscosity of the solution, resulting in augmented shear stress; second, the presence of glucose as an osmotic agent (which is not harmful to kidney) provokes acidosis in liver and pancreas cells [22]. Furthermore, as another side effect, HES increases the hyper-aggregation of erythrocytes as compared to PEG35 in flushing solutions, and this can create difficulties for suitable rinsing and preservation during MP [43,44]. Therefore, given the limitations of the current MP perfusates, we explored whether they can be improved based on previous evidences.

In this review we present and discuss the preliminary results on the advantages of using PEG35 perfusates for HOPE in liver graft preservation strategies. PEG35 seems to enhance mitochondrial protection. Moreover, previous evidence suggests the importance of viscosity, shear stress and signal transduction mechanisms and their effect on endothelial glycocalyx, and how PEG35 can modulate these processes during hypothermic liver machine perfusion.

## 2. Static Cold Storage (SCS) and PEG

SCS is based on the idea of keeping an organ in a box at a cold temperature in order to slow down its metabolism. This simple strategy has been the most widely used technique since the early beginnings of transplantation until even now, due to its simplicity and effectiveness. However, it is not ideal as does not completely halt the metabolism, as anaerobic reactions can still be found to operate. Although it is easy to handle, it has been demonstrated that it is not as effective as MP in kidney with expanded criteria donors (ECD) and donors after circulatory death (DCD) [45,46].

**Figure 1:**
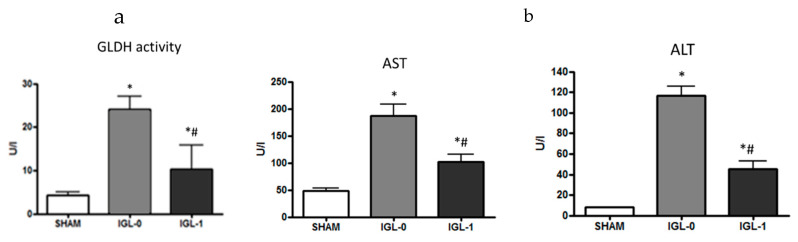
Differences between IGL-0 and IGL-1. (**a**) Mitochondrial damage as glutamate dehydrogenase (GLDH) activity. (**b**) Liver damage as transaminases aspartate aminotransferase (AST) and alanine aminotransferase (ALT). Groups are divided into SHAM (no treatment), fatty livers preserved 24 h at 4 °C in IGL-0 solution (without PEG35) and commercial IGL-1 solution. Results are expressed as mean ± SEM (*n* = 6). * *p* < 0.05 significant differences as compared to SHAM; # *p* < 0.05 as compared to IGL-0.

In previous studies, our group has seen that the presence of PEG35 in the IGL-1 solution is a key factor in mitochondria preservation and in reduction of transaminases. Figure 1 compares IGL-1, which contains PEG35 at 1 g/L), with IGL-0, which has same composition as IGL-1 but without any PEG.

Those results could partly explain the difference found in graft protection when IGL-1 and UW solutions were compared after 24 h SCS prior to reperfusion [6]. This was corroborated by a significant decrease in the transaminases AST/ALT release (liver injury) and in GLDH enzyme activity (mitochondrial damage). The absence of oxygen in SCS forces cells to switch to an anaerobic metabolism, which leads to an energetic breakdown, provoking cellular edema secondary to membrane imbalance, a drop in the pH and accumulation of subproducts that later on greatly contribute to ROS formation and general organ damage. Inability to generate sufficient ATP to maintain ionic homeostasis affects the integrity of the mitochondria. All these processes can be modulated by the preservation solution during cold storage in order to maintain the graft viability; for this, the presence of PEG35 is determinant, as we have previously demonstrated [47]. The presence of PEG35 in the IGL-1 solution is more effective at preventing energy metabolism failure than UW, thus favoring a better graft conservation [6].

The aforementioned energetic breakdown of the cell provokes a mitochondrial depolarization and the closing of mitochondrial permeability transition pore (MPTP), which is accompanied by increases in lactate, succinate and other ROS precursors associated in this oxygen deprivation condition. In this sense, mitochondrial protection conferred by PEG35 would be a critical point in ischemias, as recently suggested by Kulek et al. [48]

With its potential cytoprotective effects in mind, we evaluated the benefits of PEG35 in rinse solution for graft washout after cold preservation [19]. The presence of PEG35 in rinse solution after static preservation in UW confirmed that this polymer is directly responsible for mitochondrial preservation during cold storage, including as well the concomitant activation of cytoprotective factors such as AMP-activated protein kinase (AMPK) and presumably the inherent cytoskeleton rearrangement [19]. These data are in accordance with Chiang et al. [49], who reported that PEG35 could contribute to a better stabilization of the liver endothelial barrier and cytoskeleton when grafts are subjected to PEG35 rinse. These facts point to the potential benefits of using PEGs as oncotic agents in perfusates to reduce the deleterious effects inherent to dynamic preservation strategies.

Oxygen deprivation during IGL-1 cold storage activates a set of protective cell-signalling pathways, such as AMPK, as a self-response of the organ in front of the energetic breakdown [50]. PEG35 promotes AMPK activation, which acts on its downstream targets and leads the graft towards an energy–conserving strategy in ischemic conditions. This AMPK activation through PEG35 contribute to limiting the impact of IRI, and in this sense, PEG35 could be considered as a preconditioning agent [51,52,53].

One of the main AMPK targets is the endothelial nitric oxide synthase (eNOS), which is responsible for the generation of nitric oxide (NO), a well-known vasodilator agent that protects the liver against IRI [51,52,53]. Thus, any activation of AMPK and HIF, triggered either by intrinsic cytoprotective mechanisms of the cell or as a result of a PEG35 upregulation, would reduce the graft injury during preservation, as happens when IGL-1 solution is used [50,51]. Further, AMPK counterbalances the exacerbated microcirculatory alterations through NO production, which is specially narrowed in the livers presenting steatosis [54,55]. In this sense, PEG35-derived NO in IGL-1 solution would at least partly explain AMPK activation as well as the improved vascular resistance and function during reperfusion [6,56,57].

**Figure 2:**
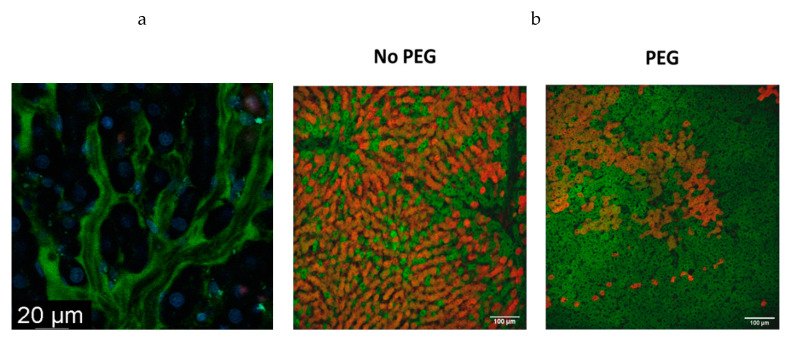
Electron microscopy imaging showing effects of PEG35 on rat liver. (**a**) Intravital microscopy of rat liver perfused with PEG35 covalently conjugated to FITC (green). Cell nuclei were labelled with intravenous injection of Hoescht 33, 342 (blue). PEG35 is deposited in the liver vascular bed. Male Sprague-Dawley rats were treated with PEG35 (10 mg/kg) and then subjected to 1 h ischemia followed by 2 h reperfusion. (**b**) Ex vivo two-photon imaging of liver grafts labelled with rhodamine 123 (green) and propidium iodide (red), showing polarized mitochondria and dead cells respectively. Liver grafts were worse preserved when they were rinsed with a solution without PEG35 (left) than with the same solution containing PEG (right). Images show better preservation of polarized mitochondria in PEG rinsed livers, as well as smaller size of cells and more compact structure, indicating that PEG, as oncotic agent, prevents cell swelling and interstitial edema.

Our recent investigations demonstrated the protective actions of intravenous PEG35 administration (10 mg/kg) in a rat model against the deleterious effects of liver IRI [25,27]. PEG35 administration was associated with NO generation and an increased protection of endothelial cell barrier, as previously evidenced in human lung endothelium by Chiang et al. [49], who reported beneficial changes due to actin rearrangements. This idea is supported by electron microscopy imaging findings, which reveal that PEG35 interacts with the luminal vascular bed when administered intravenously (Figure 2). This PEG35 deposition on the vascular endothelium could explain the subsequent activation of transduction mechanisms of protective cell signalling activation pathways, similar to those occurring in ischemic preconditioning, such as AMPK and eNOS activation in the rat liver [56,57]. All of them presumably are involved in the cytoskeleton rearrangement [19,49].

## 3. Endothelial Glycocalyx: Fluid Dynamics and PEG35 Effects

Additional factors to be considered in dynamic preservation are the physical characteristics of the perfusate, such as viscosity and shear stress, which can provoke both mechanotransduction or/and destruction of the glycocalyx (GCX) [42,58]. The GCX comprises the thin luminal sugar monolayer that covers the graft endothelia and is highly exposed to all circulating fluids, and it is damaged within human liver grafts during preservation [58,59,60].

The endothelial GCX comprises proteoglycans, glycoproteins and glycosaminoglycans. Due to its superficial location covering the endothelial cells (EC), the GCX affects the vascular permeability, mitigates blood cell-vessel wall interactions and plays a critical role in EC mechanosensing and transduction in the blood flow regulation [60,61,62].

The vasculo-protective role of GCX may be disrupted or modified by diverse pathophysiological conditions and clinical settings, such as the IRI occurring in liver transplantation (TX) [63]. The fluid dynamics (blood in the case of TX, and perfusate in the case of MP) are intrinsically related (to a greater or lesser extent) to shear stress, which in turn may induce the disruption or destruction of GCX. Shear stress is highly determined by the viscosity of the solution, which depends on the composition and the oncotic agent. Therefore, obtaining optimum shear stress associated with viscosity is a critical point for preserving GCX integrity and graft viability.

As reported by Schiefer et al. [63], the damage to the GCX is well-correlated with alterations in graft injury and function in clinical liver transplantation, and the assessment of some of its components, such as syndecan or heparan sulphate, can be used as a marker of GCX degradation. 

As shear stress undermines many aspects of the GCX, it is not less important the mechanotransduction [58,59,60,61,62]. One of the main consequences of an adequate functioning of the GCX mechanotransduction is NO production [60]; therefore, mitigating GCX destruction is a necessary step to regulating NO production in order to maintain a good perfusion and an adequate preservation [60]. In previously reported studies, we demonstrated the relevance of NO induced by PEG35, which correlated with an enhanced preservation of the GCX during static preservation when comparing two solutions, IGL-1 and HTK [64].

With these considerations, and in accordance with the observations of Schiefer et al. [63], we corroborated that the GCX suffers an alteration during hypothermic preservation [64]. The improved preservation of the GCX, supported by our data, correlate with an increased presence of NO in the group where livers were preserved in 24 h SCS with a solution containing PEG35 (IGL-1) as compared to one without oncotic agent (HTK). In this case, the protective benefits of IGL-1 were presumably associated with the presence of the oncotic agent PEG35 [64].

**Scheme 1:**
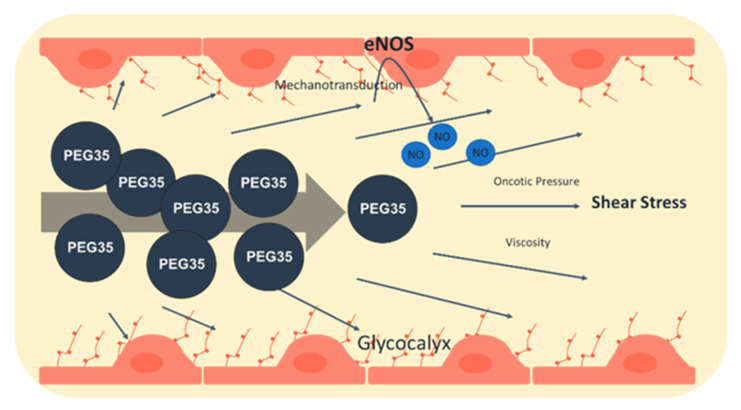
Schematic depiction of proposed PEG35 mechanotransduction through its interaction with the glycocalyx due to its physical properties that affects the sheer stress in HOPE strategies. This results in an increase of nitric oxide (NO) production through an upregulation of eNOS [60,64].

In sum, the presence of PEG35 in perfusates for MP, such as HOPE, could favour a more efficient GCX integrity, which will be translated in an increased mechanotransduction capability and generation of endothelial NO. Overall, this contributes to a better endothelial barrier protection, as shown in Scheme 1. It is important to point out that the viscosity of UW or Belzer MPS solutions with HES is twice as much as IGL-1 with PEG35 (2.4 centipoise [cP] vs. 1.2 cP, respectively). Consequently, the use of PEGs should be considered in order to confer the right viscosity and oncotic pressure features to the perfusate for the preservation of the GCX [19,44].

## 4. HOPE and PEG35 Perfusates

Machine perfusion allows the dynamic perfusion of the organs and was developed decades ago. However, the difficulties to implement the logistics kept their impact to a low profile. Nowadays, with the advance in innovation, their design is more portable and efficient. HOPE is the modality that embraces a dynamic perfusion at the range of 4–11 °C with active oxygenation of the perfusate [36,37,38,39].

MP allows a continuous supply of oxygen and nutrients while flushing cellular waste products from the liver, thereby preventing the damage cascade build-up that occurs in SCS. Furthermore, the oxygen flow permits energy production through the mitochondrial electron transport chain, helping to restore cellular homeostasis and to prevent mitochondrial collapse [65]. In addition, this technique also enables organ viability to be monitored and makes pharmacologic interventions possible.

A complete stop of oxidative phosphorylation that occurs in SCS has practical consequences not only for the ability to limit the extent of hypoxia but also for reperfusion once normal conditions are re-established, as this stop hinders cells from reaching the previous levels of production due to the destruction of the mitochondria. However, the addition of oxygen in HOPE provides a way to keep certain levels of ATP production, which as a side effect implies a lower accumulation of ROS precursors derived from anaerobic metabolism (such as succinate), giving cells a better chance to return to normality. In this sense, the presence of PEG35 in the perfusate could confer mitochondrial protection during HOPE, as evidenced by our prior studies with PEG35 rinse solution and IGL-1 [19].

In a comparative study using a rodent model of DCD liver grafts, machine perfusion strategy proved to be more protective than SCS [66]. In addition, comparison among different machine perfusion approaches (e.g., warm vs. cold perfusion) showed that normothermic oxygenated perfusion failed to protect from lethal injury in grafts exposed to 1 h warm ischemia, with a concomitant activation of Kupffer- and endothelial cells [66]. On the other hand, HOPE prevented the development of lethal graft injury, probably by the downregulation of electron transfer rates, which hampers the initial oxidative stress. These results suggest a better outcome for the HOPE technique to rescue DCD livers [66,67].

Further, a 1 h HOPE treatment after SCS protected liver grafts from initial ROS and damage-associated molecular pattern (DAMPs) release after transplantation, alongside with decrease activation of inflammatory pathways [67]. This HOPE period was also appropriate for recovering ATP loading prior to reperfusion and for reducing cell death during reperfusion [68].

The composition of perfusion solutions for hepatic hypothermic oxygenated perfusion are identical to those used for SCS. As mentioned above, the main drawback of solutions containing HES (such as Belzer MPS) is their high viscosity, which may lead to sinusoidal shear stress. To overcome these shortcomings, a new solution containing PEG35 instead of HES was developed, called Polysol [23,69]. As an oncotic agent, PEG35 exerts an oncotic pressure similar to HES but with a relatively lower viscosity and consequently less shear stress in the hepatic sinusoid. A better liver function and less liver damage was observed using the Polysol solution as compared to Belzer MPS [23,69]. In addition, 24 h of MP using Polysol showed a better preservation as compared to the same solution but with HES instead of PEG (Polysol-HES), highlighting the protective role of PEG. A lower molecular weight PEG, PEG20, supplemented to a Celsior solution offered a better protection of pig kidneys recovered after cardiac death using MP, as compared to Celsior without supplementation or to Belzer MPS [70]. Schlegel et al. [71] showed that endothelial cleaning or repair of the GCX represents an important mechanism of protection conferred to the liver by HOPE. The GCX can be cleaved by either enzymatic cleavage of the proteoglycan core proteins or direct oxidative stress from ROS underlying ischemia reperfusion injury (IRI), inducing endothelial permeability and edema [72]. As mentioned above, Lopez et al. [64] showed compelling evidence for the benefits of IGL-1 solution on steatotic livers, highlighted by the importance of GCX protection during SCS. It is known that PEG35, present in IGL-1 solution, prevents cell swelling and vascular endothelial damage through the stabilization of lipid membranes and by lowering membrane permeability. To deepen the findings of Lopez et al. [64] regarding the role played by GCX during hepatic IRI and its connection to PEG35, the shear stress should be also taken into account. HOPE seems like a valid strategy to investigate the shear stress inherent in IRI and its effect on the GCX integrity using a perfusate containing PEG35. Such a therapeutic approach of GCX protection could potentially enhance organ viability of marginal grafts and diminish the severity of IRI.

**Figure 3:**
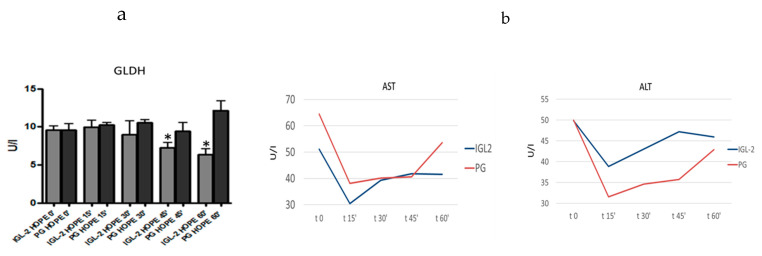
Comparison of Belzer MPS and IGL-2 in HOPE. (**a**) Mitochondrial damage as GLDH activity in fatty livers preserved for 7 h at 4 °C and analyzed for 1 h (measured in 15 min intervals) of HOPE in IGL-2 solution (PEG35-enriched IGL-1 solution) and Perf-gen solution. (**b**) AST/ALT levels during HOPE. Significant differences were found in mitochondrial damage at 40 min and 60 min of HOPE between the two solutions. However, this did not affect AST/ALT levels during the same period. * *p* < 0.05 represent significant differences vs. Perf-gen group (PG-HOPE).

Based on these strong observations, our target was to ascertain whether PEG could have a relevant effect in protecting the liver during HOPE at the mitochondrial level. For this reason, we compared the only available perfusates Belzer MPS and a new one including PEG35 (IGL-2) (Table 2) for 1 h of HOPE after 7 h of SCS in the same solutions, using liver in Sprague Dawley rats [24]. Table 2 shows the composition of the two solutions tested. The comparative levels of mitochondrial damage (measured as GLDH activity) in both MP solutions revealed a significant prevention of mitochondrial damage IGL2 solution in HOPE conditions (Figure 3a), although no significant differences were found in transaminases levels (Figure 3b).

To measure the deleterious effects of IRI, other markers has been suggested, such as the mitochondrial enzyme ALDH2 [73] and glycocalyx [74]. We observed an increase in ALDH2 activation in liver grafts that had been subjected to HOPE followed by 1 h of normothermic reperfusion (unpublished data). This ALDH2 activation is a protective factor that would prevent lipoperoxidation subproducts, such as 4HNE, associated with IRI [73]. In this context, improvement of the mitochondrial status during HOPE is closely linked to the presence of PEG35 [75], and it could be a critical point for restoring the protective mitochondrial mechanisms and modulating succinate accumulation during cold preservation [76,77,78].

In the hypothermic oxygenated perfusion (HOPE), having a continuous oxygen delivery allows cells to maintain and restore the basal mitochondrial machinery as compared to the static preservation (no oxygen support) [36,38]. These HOPE benefits were more potentiated in IGL2-perfusate (containing PEG35) than in generic Belzer perfusate (containing HES). The better mitochondrial protection was concomitant with an increase in mitochondrial ALDH2 activity measured 1 h reperfusion after (unpublished data). In any case, the cold cellular oxygenation during HOPE using PEG35 perfusate is likely to help reduce accumulation of some metabolites (e.g., succinate) that are responsible for dysfunctional liver graft mitochondria as compared to static preservation [77,78]. Thus, the clearance of such metabolites by the dynamic flow observed in HOPE might be an important way to guarantee proper mitochondrial function during early normothermic reperfusion.

## 5. New Biomarkers for Dynamic Preservation

**Scheme 2:**
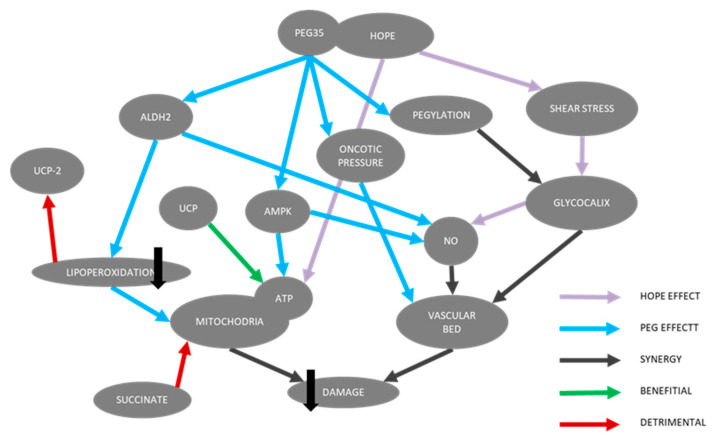
Schematic representation of the working mechanisms of PEG35 and HOPE leading to improvement of the mitochondria and the vascular bed and thereby prevent damage. PEG35 upregulates ALDH2, which prevents lipoperoxidation. High levels of lipoperoxidation promotes UCP-2 formation. However, reduction of lipoperoxidation prevents the formation of ROS in the mitochondria. The upregulation of AMPK exerted by PEG35 has a double side effect: it promotes NO formation, which helps vasodilation, and prevents the energetic breakdown of the mitochondria. In this sense, upregulation of UCP also prevents this depletion of ATP. Furthermore, succinate has been reported as a subproduct of the anaerobic metabolism, making it is a reliable marker of the status of the mitochondria.

Widely known parameters of clinical relevance, such as transaminases and lactate, are indicative but not definitive for liver status. Therefore, another scope of this study could be the assessment of other markers that could bring a more accurate diagnosis of the status of the liver. For instance, it might be of interest to assess levels of syndecan and heparan sulphate, which would give us an insight of the status of the GCX in HOPE strategies [63,64,65,66,67,68]. Further, it would be interesting to assess mitochondrial parameters besides ALDH2 [47], such as succinate [77,78], or others that have been recently linked both to lipoperoxidation and energetic levels, such as UCP1 and UCP2 [79,80,81,82,83], which could provide relevant information, especially when steatotic livers are used (Scheme 2). The assessment of these new proposed markers involved in different critical points of different crucial pathways brought together could strengthen the informative value of the most widely used, such as transaminases.

## 6. HOPE-COR-NMP and PEG35

Considering all the data presented above, there is a strong evidence that PEG35 inclusion in a perfusate for HOPE (such as IGL-2 [24]) could be a promising tool to be applied to other modalities of graft preservation, such as HOPE-COR-NMP, which was recently used in clinical settings [84].

PEG35 is a suitable candidate to increase preservation quality of liver grafts subjected to any modality of dynamic preservation, such as HOPE or combined methodologies such as HOPE-COR-NMP, due to three main points: 1) PEGs are stable, nontoxic, non-immunogenic and water-soluble molecules, as recognized by FDA [11]; 2) PEG35 confers oncotic and cytoprotective properties to be used for liver transplantation as defined by ELTR [9]; and 3) PEG35 is protective against IRI in experimental hypothermic, normothermic and hyperthermic conditions [26,27,85,86].

Given the promising results of PEG-based solutions in HOPE, it seems justified to explore its use in promising combination techniques such as HOPE-COR-NM, keeping in mind the physical characteristics of non-Newtonian fluids at different temperatures that make that the viscosity increases with the decrease in temperature. Therefore, as the viscosity of IGL-2 at 4 °C seems to be suitable for HOPE, increasing the temperature to subthermic and normothermic conditions will only lower the viscosity. Such levels of viscosity would not be a problem for normothermic MP. We therefore believe that it is of great interest to assess PEG potential benefits in the mitochondria status, the GCX and the mechanotransduction processes inherent to graft preservation in any dynamic condition [6,23,24].

## 7. Concluding Remarks

This newly proposed perfusion strategy might have an especially relevant impact in those points where dynamic perfusion and PEG35 act in synergy in critical cell survival and graft integrity points, such as the mitochondria and the GCX. In this sense, any improved aspects during the ischemic time will positively affect the reperfusion phase, with an emphasize on rescuing marginal grafts, such as fatty livers for liver transplantation.

Along these lines, we report a new window of MP strategy improvements focused on the exploration of new perfusates with adequate physical characteristics that could confer both cytoprotection and mitochondrial restoring [75]. One tool to do so is to incorporate agents like PEG35 into HOPE perfusates; this is in contrast to HES, which is present in Belzer MPS solutions and which can cause a potential aggregation of resting red blood cells and endothelial damage due its high viscosity that increases shear stress. The use of PEG35 in perfusates could be a rational and potential option to be evaluated in promising strategies such as HOPE-COR-NMP [84] for rescuing liver grafts for transplantation.

More studies are needed to deepen our comprehension of the dynamic systems as compared to the static ones, and specifically their relationship with mitochondrial preservation, as well as the improvement of perfusates at both the molecular and physical levels. This is likely to represent one of the milestones of organ preservation in the near future, where the challenge is to take advantage of marginal organs, to increase donor pool for transplantation.

## Figures and Tables

**Table 1:** PEG-based preservation solutions.

Preservation Solution	PEG (kB)	Concentration (g/L)
IGL-1	35	1
IGL-2	35	5
Polysol	35	20
SCOT	20	15

**Table 2:** Composition of IGL2 and Belzer MPS solutions.

Preservation Solution	Belzer-MPS	IGL-2
Electrolytes (mmol/L)		
K +	25	25
Na +	120	125
Mg2 +	5	5
SO4 2-	5	5
Ca +	0.5	
Zn 2+		0.091
Buffers (mmol/L)		
Phosphate	25	25
HEPES	10	
Histidine		30
Impermeants (mmol/L)		
Mannitol	30	60
Lactobionic acid		80
Dextrose	10	
Ribose	5	
Gluconate	85	
Colloids (g/L)		
Hydroxyethyle starch	50	
Polyethylene glycol-35		5
Antioxydants (mmol/L)		
Glutathione	3	9
Metabolic precursors (mmol/L)		
Adenosine		5
Adenine	5	
NaNO2 (nmol/L)		50
pH	7.4	7.4
Osmolarity (mosmol/L)	320	320
Viscosity (cP)	2.4	1.4

## Abbreviations

4H NE	4-Hydroxynonenal
ALDH2	Aldehyde dehydrogenase-2
ALT	Alanine aminotransferase
AMPK	AMP-activated protein kinase
AST	Aspartate aminotransferase
Belzer-MPS	Belzer Machine Perfusion Solution
DAMPs	Damage associated molecular patterns
DCD	Donor after cardiac death
EC	Endothelial cells
ECD	Extended criteria donors
eNOS	Endothelial nitric oxide synthase
GCX	Glycocalyx
GLDH	Glutamate dehydrogenase
PEG	Polyethylene glycol
HES	Hydroxyethyl starch
HIF	Hypoxia inducible factor
HOPE	Hypothermic oxygenated perfusion
HTK	Histidine-tryptophan-ketoglutarate
i.v.	Intravenous
IGL-1	Institut Georges Lopez 1
IRI	Ischemia–reperfusion injury
MP	Machine perfusion
MPTP	Mitochondrial permeability transition pore
NO	Nitric oxide
ROS	Radical oxygen species
SCS	Static cold storage
SS	Shear stress
TX	Liver transplantation
UCP1	Uncoupling protein 1
UCP2	Uncoupling protein 2
UW	University of Wisconsin
